# A New TASK for Dipeptidyl Peptidase-like Protein 6

**DOI:** 10.1371/journal.pone.0060831

**Published:** 2013-04-09

**Authors:** Brian M. Nadin, Paul J. Pfaffinger

**Affiliations:** Department of Neuroscience, Baylor College of Medicine, Houston, Texas, United States of America; Georgia State University, United States of America

## Abstract

Dipeptidyl Peptidase-like Protein 6 (DPP6) is widely expressed in the brain where it co-assembles with Kv4 channels and KChIP auxiliary subunits to regulate the amplitude and functional properties of the somatodendritic A-current, I_SA_. Here we show that in cerebellar granule (CG) cells DPP6 also regulates resting membrane potential and input resistance by increasing the amplitude of the I_K(SO)_ resting membrane current. Pharmacological analysis shows that DPP6 acts through the control of a channel with properties matching the K_2P_ channel TASK-3. Heterologous expression and co-immunoprecipitation shows that DPP6 co-expression with TASK-3 results in the formation of a protein complex that enhances resting membrane potassium conductance. The co-regulation of resting and voltage-gated channels by DPP6 produces coordinate shifts in resting membrane potential and A-current gating that optimize the sensitivity of I_SA_ inactivation gating to subthreshold fluctuations in resting membrane potential.

## Introduction

The cerebellum plays a critical role in motor learning and cognitive function by adjusting neuronal firing patterns to match desired behavioral outcomes [1]. Cerebellar granule (CG) cells are the most abundant neuron type in the human body and are responsible for forming the parallel fibers of the cerebellum that provide one of the two main excitatory inputs to Purkinje neurons. Factors regulating CG cell firing are critically important for the function of the cerebellum since more than 150,000 different CG cell inputs converge onto a single Purkinje neuron dendrite [2]. Precise regulation of both resting and voltage-gated channels is required for CG cells to process input signals and generate appropriate output firing. However, cellular mechanisms that underlie the coordinate regulation of resting and voltage-gated channels are poorly understood.

Dipeptidyl peptidase-like protein 6 (DPP6) is an inactive protease homolog that is an important regulator of CG cell excitability. DPP6 was identified as an important component of Kv4 based channels following co-purification from cerebellum [3]. Heterologous expression studies have shown that DPP6 acts in coordination with KChIP auxiliary subunits to regulate the expression level and functional properties of Kv4 based ion channels [3], [4], [5]. In mouse CG cells, using a lentiviral based RNAi knockdown and rescue strategy, DPP6 was shown to be required to properly form the Kv4 dependent somatodendritic A-type current (I_SA_) [6]. Interestingly, knocking down DPP6 also dramatically changes the resting membrane properties of CG cells by increasing the resting input resistance. Although “window” currents from A-type channels can contribute to the regulation of resting input resistance [7], other potential mechanisms exist by which DPP6 expression could affect excitability. In CG cells, I_K(SO)_, a muscarine sensitive resting membrane potassium current plays an important role in regulating excitability [8]. Previous work has suggested that I_K(SO)_ is most likely formed by K_2P_ type channels, and may in fact be an amalgamation of several different K_2P_ channel types [9], [10], [11], [12].

K_2P_ channels have emerged as a very important class of channels controlling background or “leak” potassium conductance in many cell types [13]. Structurally K_2P_ channels dimerize to form a pore structure that is very similar to other potassium channels due to the presence of two P-loop domains within each subunit [14], [15]. Previous studies on I_K(SO)_ have indicated the likely importance of the TASK type K_2P_ potassium channels in forming I_K(SO)_ [10], [12], [16], [17]. Similar to I_K(SO)_, TASK-1 and TASK-3 conductances are outwardly rectifying, suppressed by muscarine and likely form functional heteromultimers in many systems [12], [18]. TASK channels are named due to their high sensitivity to lowered pH (**T**WIK related **A**cid **S**ensitive **K** ^+^ channel) [19], and can be differentiated from other muscarine or acid sensitive conductances by their relative sensitivity to other blockers such as extracellular Zn^2+^ [16].

The focus of this study was to determine the mechanism by which knockdown of DPP6 results in such dramatic changes in the excitability of CG cells. Here we report that in addition to regulating I_SA_, DPP6 expression is also required to produce normal levels of I_K(SO)_ in CG cells. Pharmacology and heterologous expression studies show that a regulation of the K_2P_ channel TASK-3 by DPP6 likely underlies this change in I_K(SO)_. We propose that co-regulation of I_SA_ and I_K(SO)_ by DPP6 may be part of an information processing system in the brain that optimally couples I_SA_ channel inactivation to the preceding pattern of subthreshold membrane potential fluctuations.

## Materials and Methods

### Electrophysiological Methods and Data Analysis

Voltage clamp to isolate I_SA_ and current clamp recordings were performed on cultured CG cells as described previously [6]. For current clamp recordings pipettes were backfilled with internal solution containing (in mM): 120 K-gluconate, 20 KCl, 10 HEPES, 0.2 EGTA, 2 MgCl_2_, 4 Mg·ATP, 0.3 Tris·GTP, 5 phosphocreatine-2Na^+^, pH 7.3. Current clamp external solution contained (in mM): 125 NaCl, 2.5 KCl, 25 NaHCO_3_, 2 CaCl_2_, 1 MgCl_2_ 10 glucose. Reported potentials are adjusted by −14 mV from the recorded membrane potentials to compensate for electrode junction potential. For pharmacological block experiments, pharmacological agents were dissolved in a modified external solution pH buffered by HEPES containing (in mM): 125 NaCl, 2.5 KCl, 10 HEPES, 2 CaCl_2_, 1 MgCl_2_, 10 glucose, pH 7.4. Zn^2+^ was added to a final concentration of 500 µM, muscarine was added to a final concentration of 10 µM and pH 6 buffer adjusted with HCl, respectively, for each experimental block condition. External blocking solutions were applied using pulled narrow-bore borosilicate glass connected to a PicoSpritzer III (Parker Hannifin Corp., Mayfield Heights, OH) at pressures below 4 psi. Solutions were applied for at least 30 s before initiating recording of the block condition.

### Molecular Methods and Primary Cell Culture

Lentiviral vectors used in this study were described previously [6]. As described previously [6], the Control vector for these studies is identical to the mDPP6 RNAi vector only with a nonspecific RNAi sequence in place of the mDPP6 RNAi target sequence. In our previous work, mDPP6 RNAi was found to be selective for mouse DPP6 and to not affect the expression of human or rat DPP6 [6]. To establish the specificity of mDPP6 RNAi effects, rescue was performed with a mDPP6 RNAi lentiviral vector co-expressing rat DPP6 (rDPP6K, which is normally expressed in CG cells [5], was used unless specifically indicated otherwise). Heterologous expression studies were performed with mDPP6K. Mouse CG cell cultures were prepared as described previously [6]. All CG cell experiments were conducted at 14–18 days *in vitro* at which time the neurons show strong repetitive firing. Some Preliminary studies were performed at 8–10 days *in vitro* [6], but we found that neurophysiological properties are changing at this age and don’t become stabilized until two weeks in culture. By that time, resting membrane properties and neuronal firing patterns stabilize and become more similar to *in vivo*, and the conductance changes in response to mDPP6 RNAi are much greater.

Mouse TASK-3 (mKCNK9) was cloned by PCR from a mouse cerebellum cDNA library generated from post-natal day 7 C57/B6J pups using a 5′ UTR primer (5′ TTAGCATCTCCTTCTTCGCGG 3′) and 3′ UTR primer (5′ TGCATGACAAATGATTCTTCC 3′). HA-mKCNK9 was generated by PCR using a 5′ primer incorporating the HA-tag (5′ TTTGAATTCGCCACCATGTA TCCATATGACGTCCCAGACTATGCTATGAAGCGGCAGAAC 3′) and a mKCNK9 internal primer (5′ TCTTCGGCTTTAAGTTTCTCC 3′). HA-DPP6K was generated by PCR using a 5′ primer incorporating the HA-tag (5′ TTTCAATTGGCCACCATGTA TCCATATGACGTCCCAGACTATGCTATGAAGGAAAAGGCA 3′) and a DPP6k internal primer (5′ TTGTTTCAACATTCCGCAGTA 3′).

For analysis of expression of K_2P_ channel variants in CG cell cultures, we used qRT-PCR with Fast SYBR Green Master Mix (Applied Biosystems) on a PTC-200 thermal cycler (MJ Research). Intron spanning primers against K_2P_ channel subunits that are expressed in cerebellum at a similar age were used [20]: TASK-1, forward (5′ AGGAGCTGGAGCGCGTCGTGC 3′), reverse (5′ ACCTTGCCTCCGTCCGTGCTG 3′); TASK-3, forward (5′ AGTCTGAGCCCCACCGCGCTG 3′), reverse (5′ CCAGCATCAGTTCCAGGTGCA 3′); TREK-1 forward (5′ GCCCAGCATGCCTGCGTCAAC 3′), reverse (5′ GGATAATCCCTGCGTTTATTG 3′); TREK-2 forward (5′ CATCTGTGTGAGTCCCCAGGA 3′), reverse (5′ GACTCCCGCGTTATCAGCATC 3′), TWIK-1 forward (5′ TGGAATTGGGACTTCACCTCG 3′), reverse (5′ CCATCTGACAGGGGCACCGTG 3′); TWIK-2 forward (5′ TCGCCAGCACGCTTGTCACCA 3′), reverse (5′ CATCTGTCAGTGGGGTCGTGT 3′). qPCR reactions were verified by melting curves, amplicon agarose gel electrophoresis and amplicon TA cloning and sequencing. All constructs were verified by DNA sequencing.

### Heterologous Expression Studies

CHO cells were maintained in DMEM supplemented with 10% fetal bovine serum, penicillin/streptomycin and L-proline. CHO cells were transfected in a 24-well plate format utilizing Lipofectamine 2000 (Invitrogen) and 1 µg total DNA (either GFP 1 µg; mKCNK9 200 ng, GFP 800 ng; mKCNK9 200 ng, DPP6K 300 ng, GFP 500 ng). Voltage-clamp recordings in CHO cells were performed 24 hours post-transfection. HEK cells were cultured as described [6] and transfected utilizing Lipofectamine 2000.

### Immunprecipitation and Western Blotting

24 hours post transfection cells were homogenized in sucrose buffer (in mM, 320 sucrose, 1 EDTA, 5 TRIS, pH 7.4, supplemented with protease inhibitor cocktail (PIC; Sigma). Homogenate was cleared by a 10 minute spin at 500 rcf and membranes isolated by high-speed centrifugation at 25,000 rcf for 40 minutes. Membrane pellet was resuspended and solubilized for 1 hr at 4°C in TNE buffer (in mM: 50 TRIS, 150 NaCl, 1 EDTA, pH 7.4, 1% TritonX-100) plus PIC. Solubilized membrane fraction was cleared with mouse IgG agarose beads (Sigma) for 2 hr at 4°C. Cleared membrane fractions were incubated for 16 hours at 4°C with monoclonal anti-HA agarose conjugate (clone HA-7, Sigma). Bound complexes were washed four times with TNE buffer and eluted by incubating in Laemmli sample buffer (BioRad) containing 100 mM DTT and PIC at 56°C for 5 minutes. Solubilized protein samples were separated by SDS-PAGE gradient gels (Invitrogen). Due to additional salts and non-ionic detergents in the L and F lanes that are no longer present in the beads, the HA sample sometimes runs slightly faster than other samples. Western blots were performed by transferring onto a PVDF membrane and probing with anti-DPP6 antibody (ab41811, 1∶1000, Abcam) or anti-KCNK9 antibody (APC-044, 1∶500, Alomone labs, Jerusalem, Israel) and detected with HRP conjugated secondaries using PicoSignal ECL (Pierce). TASK-3 protein predominantly runs in our gels at higher molecular weights than predicted for the free monomer, possibly due to aggregation, as has been seen by others [21]. Initial Westerns were performed using film, but later Westerns were exposed digitally using an ImageQuant LAS4000 (GE). Sample loading amounts compared to “L” load are: HA: 2x, Flow thru: 0.25x. Western quantification was performed using ImageQuant software with “rubber band” background subtraction. To determine the efficiency of the co-IP, we measured the fraction of the Load protein that was immunoprecipitated depending upon whether the protein was tagged or not. The ratio of the fraction immunoprecipitated indirectly when untagged divided by the fraction directly immunoprecipitated when tagged is the Co-IP ratio. This ratio corrects for systematic inefficiency in the immunoprecipitation and detection protocol.

### Data Analysis and Statistics

Data analysis was performed using Clampfit10 (Molecular Devices), OriginPro7 (OriginLab), and Excel (Microsoft). Statistical analyses were performed using OriginPro7 and Analyse-it for Excel (Analyse-it) and on-line servers (http://www.graphpad.com/quickcalcs/index.cfm; http://elegans.som.vcu.edu/~leon/stats/utest.html). Data sets were not significantly different from normality using the Shapiro-Wilks test. P values were determined using both independent two-tailed t-test and the non-parametric Mann Whitney U test, both of which gave the same results for significance testing at our alpha level of P = 0.05. Reported P values are from t-test. I–V curves were compared using ANOVA on the multiple regressions with OriginPro7. All comparisons reported were significantly different unless otherwise indicated by NSD (Not Significantly Different). I–V curves were compared by one-way ANOVA on the voltage step series. Modeling of DPP6 sensitive current pharmacology was done with the function: y = P_s_(x−N_s_)/x, where x is the resting conductance, y is the fraction of the resting conductance that is blocked by the treatment, N_S_-Total mDPP6 RNAi insensitive rest conductance, and P_S_ is the sensitivity of TASK-3 channels to the treatment. N_S_ was fixed at 1.1 nS based on the average G_rest_ for mDPP6 RNAi treated neurons. P_S_ was determined by non-linear least squares fit to the data and is indicated by the dashed line. Membrane corner frequency (f_c_) was calculated as f_c_ = 1/(2πτ_m_). Power spectra and RMS values were calculated by subtracting the average signal from the record to obtain fluctuations around zero. Power Spectra were calculated by FFT using OriginPro. RMS was calculated by taking the square root of the average for the square of the fluctuations around 0. Inactivation sensitivity was calculated by dividing the channel Inactivation standard deviation (in %) by the standard deviation for the membrane potential fluctuations.

### Computational Modeling

CG cell modeling was performed using a single 12.5 µm diameter compartment NEURON model [6], [22]. A stochastic synaptic current noise generator previously developed by Destexhe et al, 2001 [23] was added to examine the effects of synaptic noise on channel gating. Excitatory and inhibitory fluctuations were varied by specifying the average conductance (G_e_, G_i_), standard deviation (SD_e_, SD_i_). Time constants (τ_e_ = 2.728 ms, τ_i_ = 10.49 ms) and zero current potential (E_e_ = 0 mV, E_i_ = −85 mV) were not varied. Both Control and mDPP6 RNAi CG cell models used the Na^+^ channel (G_Na_ = 0.1 S/cm^2^), Ca^2+^ channel (G_Ca = _0.6 µS/cm^2^), Kv channel (G_K = _0.18 mS/cm^2^) and K_Ca2+_ (G_KCa_ = 1.5 mS/cm^2^) channel models previously developed for CG cells [6], [24].To better describe the multiple exponential components found in the gating of the native I_SA_ channel we switched from H&H type models to multi-state models developed using QuB [25]. The Kinetic model for I_SA_ following mDPP6 RNAi treatment (G_ISA(R)_) only allows for inactivation through closed states. The Control condition I_SA_ model (G_ISA(C)_) has accelerated kinetics and adds additional states for N-type inactivation produced by DPP6a [26] and a deeper closed inactivation state produced by DPP6K [5]. Fits of these models to the steady state and kinetic properties of native channels I_SA_ is shown in Figure S1. TASK-3 (G_TASK-3_) was modeled with two independent gates as described previously [27]. The mDPP6 RNAi resistant K_2P_ current (G_K2P_) was identical except one gate was made slightly faster and more voltage dependent to better fit the resting current following mDPP6 RNAi treatment. A small Na^+^ selective linear leak component (G_L_) was added to account for a resting potential more positive than E_K_. The Control CG model added G_ISA(C)_, G_TASK-3_, G_K2P_, and G_L_. The mDPP6 RNAi CG model added G_ISA(R)_, G_K2P_, and G_L_. Reversal potentials were set at: E_Na_ = 60 mV, E_K_ = −91.5 mV.

### Ethics Statement

The procedures on animals conducted in this work were performed in strict accordance with Animal Welfare Act, the Public Health Services Animal Welfare Policy, and The National Institute of Health Guide for Care and Use of Laboratory Animals. The experimental protocol was approved by the Institutional Animal Care and Use Committees (IACUC) of Baylor College of Medicine (Protocol Number: AN-752). Following the approved protocol, every effort was made to minimize suffering.

## Results

### DPP6 Regulates the Excitability of Cerebellar Granule Neurons Independent of Kv4.2 Channels

**Figure 1:**
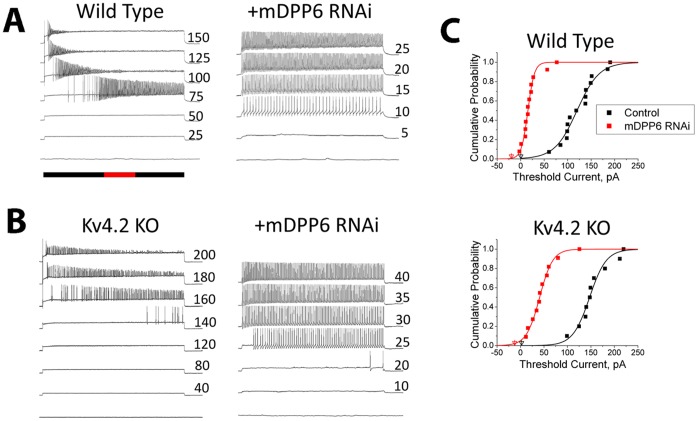
DPP6 regulates excitability of CG cells independent of its control of Kv4 channels. Mouse CG cells were recorded by current clamp following infection with a lentiviral vector expressing EGFP (control) or EGFP plus mouse DPP6 RNAi (+mDPP6 RNAi). **A)** Sustained current injections, duration indicated by black bar, were given at the level indicated to the right of the trace (pA) to elicit action potential firing (Red bar indicates 1 s). Following treatment with mDPP6 RNAi, threshold current is reduced and cells fire in response to much smaller current injections. **B)** Threshold current in CG cells from Kv4.2 KO animals is similar to wild type despite the large reduction in I_SA_ amplitude. Threshold current in CG cells from Kv4.2 KO animals remains sensitive to mDPP6 RNAi showing that the regulation of threshold by DPP6 is independent of the level of I_SA_. **C)** Current required for initiation of action potentials under different conditions is plotted as a cumulative distribution plot. Holding current indicated by the triangles was applied to set a common resting potential of −85 mV. In CG cells from both Wild Type (n = 14) and Kv4.2 KO (n = 10) animals, treatment with mDPP6 RNAi (WT, n = 13; Kv4.2 KO, n = 11) significantly reduces the current required for action potential initiation (WT,Kv4.2 KO: P<0.0001) by around 100 pA.

Application of a lentiviral vector expressing RNAi targeting a mouse DPP6 (mDPP6) core sequence dramatically increases the excitability of mouse CG cells. In Fig. 1A we show a representative CG cell current clamp recording where the neurons have been infected with a lentiviral vector expressing only EGFP (Wild Type). On average such wild type recordings require 125±10 pA of current injection to reach threshold. In contrast, if the same neurons are infected with a lentiviral vector also expressing mDPP6 RNAi (+mDPP6 RNAi), the neurons begin to fire in response to much smaller current injections (+mDPP6 RNAi: 22±6 pA (P<0.0001)). To determine if these effects are mediated through changes in I_SA_ “window” currents [7] or are an unexpected consequence of manipulating DPP6 protein levels, we sought to disrupt I_SA_ while still preserving DPP6. Knockout of the KCND2 gene (Kv4.2 KO) eliminates Kv4.2 protein and reduces I_SA_ in CG cells by approximately 80%, similar to mDPP6 RNAi [6]. Interestingly, DPP6 protein levels remain high in Kv4.2 KO neurons, although KChIPs, another class of I_SA_ auxiliary subunit protein, are almost completely lost when Kv4.2 is knocked out [6], [28], [29], [30], [31]. The Kv4.2 KO mice therefore provides an interesting way to potentially distinguish between DPP6 effects mediated through I_SA_ and those that are not.

As the representative recording in Fig. 1B shows, CG cells from Kv4.2 KO animals (Kv4.2 KO) have a firing threshold similar to that of wild type CG cells (Kv4.2 KO: 156±12 pA, NSD, P = 0.055). We next tested whether threshold current in these Kv4.2 KO CG cell remain sensitive to mDPP6 RNAi. The representative recordings from a Kv4.2 KO CG cell treated with mDPP6 RNAi (+mDPP6 RNAi, Fig. 1B) shows that despite the large reduction of I_SA_ in the cells threshold current drops dramatically (+mDPP6 RNAi: 49±10 pA (P<0.0001)). Cumulative distribution functions for the current required to reach threshold were computed for the different conditions (Fig. 1C). Although there is consistently a slightly higher threshold current (∼20 pA) for Kv4.2 KO compared to wild type treated under similar conditions (possibly reflecting some compensatory changes in the knockout [32]), mDPP6 RNAi treatment reduces threshold current by about 100 pA in CG cells from both wild type and Kv4.2 KO animals (WT: 96±13.6 pA, Kv4.2 KO: 108±15.3 pA, NSD (P = 0.58)). We therefore conclude that the effect of DPP6 on threshold current is independent of the level of I_SA_ and likely involves an alternate regulatory pathway.

### DPP6 Controls Excitability by Modulating the Level of I_K(SO)_ in CG Cells

**Figure 2:**
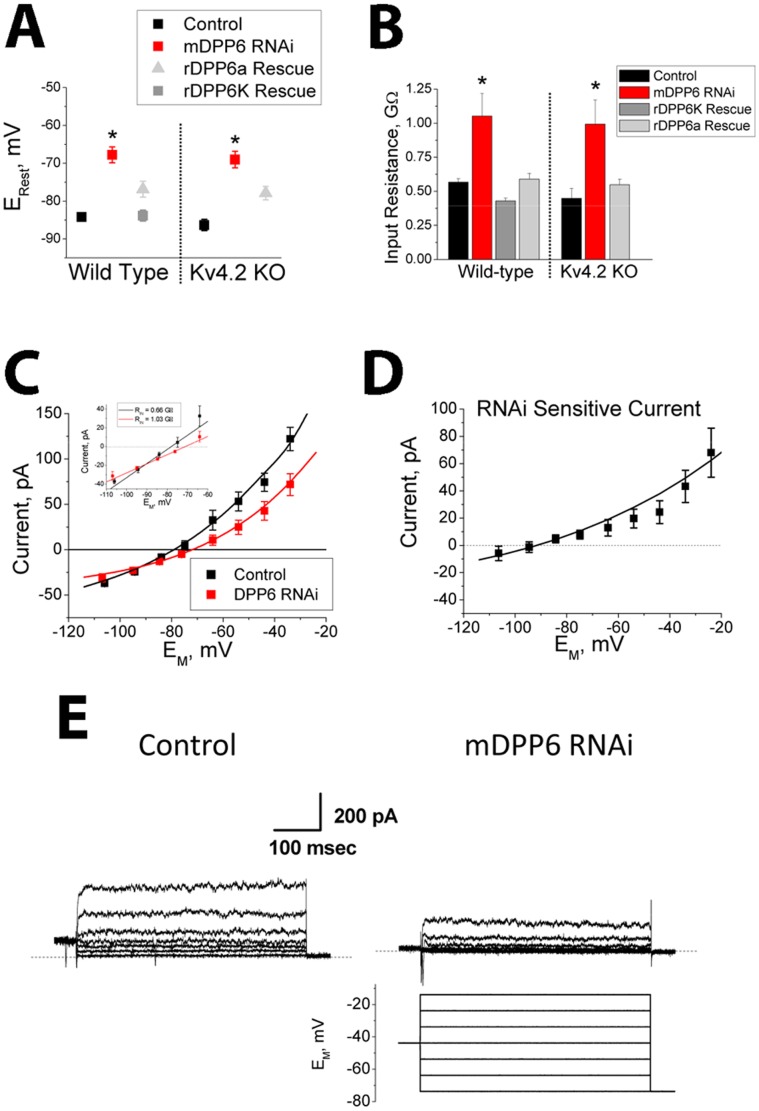
DPP6 regulates an outwardly rectifying resting membrane potassium conductance. CG cells were treated with lentiviral vectors expressing EGFP (control: WT (n = 32); Kv4.2 KO (n = 9)) or mDPP6 RNAi (WT (n = 13), Kv4.2 KO (n = 6)) to knockdown DPP6. For rescue experiments the lentiviral vectors also co-expressed rat DPP6 (rDPP6), which is insensitive to the mouse DPP6 RNAi due to coding differences in the RNAi target sequence. Both rDPP6K and rDPP6a splice variants, which are highly expressed in CG cells, were tested (rDPP6K: WT (n = 32); rDPP6a: WT (n = 31), Kv4.2 KO (n = 4)). **A)** Knockdown of mDPP6 causes a depolarization of the resting membrane potential which is reversed by co-expression of rDPP6. Results for wild type and Kv4.2 KO animals are indistinguishable (NSD: control (P = 0.33); mDPP6 RNAi (P = 0.72); rDPP6a (P = 0.85)). (data plotted ± SEM). **B)** Resting input resistance is increased for CG cells following RNAi treatment and this effect is reversed by co-expression of rDPP6. Results between CG cells from control and Kv4.2 KO animals are indistinguishable (NSD: control (P = 0.15); mDPP6 RNAi (P = 0.74); rDPP6a (P = 0.72)). (data plotted ± SEM). **C)** Average membrane current versus membrane potential for control (n = 24) and mDPP6 RNAi (n = 13) treated CG cells. Knockdown of DPP6 reduces the amplitude of an outwardly rectifying current (P = 0.0002). Smooth curves were generated with NEURON using the following conductances (**mDPP6 RNAi:** G_K2P_ = 60 nS; G_L_ = 0.133 nS; **Control:** Same+G_TASK-3_ = 30 nS). Inset expands the axes near the resting membrane potential to show that a linear Ohmic fit to this current can explain the change in input resistance (Slope change) and E_rest_ (0 current potential shift). (data plotted ± SEM). **D)** Difference current for mDPP6 RNAi treated cells compared to control shows that the RNAi sensitive current is outwardly rectifying and reverses near −90 mV as expected for a potassium selective conductance. (data plotted ± SEM). Smooth curve shows TASK-3 model fit to the data (G_TASK-3_ = 30 nS). **e)** Representative voltage clamp traces show that the conductance regulated by mDPP6 RNAi is outwardly rectifying similar to I_K(SO)_ conductance previously described in CG cells. Scale bar: 200 pA, 100 ms.

Biophysical analysis of CG cells treated with mDPP6 RNAi reveals a significant positive shift in resting membrane potential (P<0.0001) (Fig. 2A) and a significant increase in input resistance (P<0.0001) (Fig. 2B). Because membrane capacitance remains stable, this suggests that DPP6 increases the level of a resting membrane conductance. To confirm that these RNAi effects are due to the specific loss of DPP6, we performed rescue experiments by adding a rat DPP6 (rDPP6) expression cassette to our mDPP6 RNAi expressing lentiviral vectors. Our previous studies showed that rDPP6 is resistant to mDPP6 RNAi due to alternative codon usage in the RNAi target sequence [6]. We tested for rescue with two rDPP6 N-terminal variants, rDPP6a and rDPP6K, which are co-expressed at high levels in CG cells [5], [33], [34]. Both variants were able to rescue resting membrane potential and input resistance (compared to mDPP6 RNAi: E_Rest_, rDPP6a (P = 0.01), rDPP6K (P<0.0001); Input Resistance, rDPP6a (P<0.0001), rDPP6K (P<0.0001)), although rDPP6K rescue was generally significantly more robust (compared to rDPP6a: E_Rest_ (P = 0.008), Input Resistance (P = 0.0009)) and was used for all subsequent rescue experiments unless otherwise stated (Fig. 2A, B). As predicted by our previous results, CG cells from Kv4.2 KO animals are similar to wild type in resting membrane potential and input resistance, and both properties remain equally sensitive to mDPP6 RNAi treatment and rescue (Fig. 2A, B). Based on these observed effects we hypothesized that the excitability changes produced by DPP6 knockdown could be due to the specific loss of a resting membrane potassium conductance which would normally drive the resting membrane potential to more negative values.

To better characterize the DPP6 dependent conductance, a series of experiments was performed to compare the sustained membrane current versus voltage relationship between control and mDPP6 RNAi treated CG cells. As shown in Fig. 2C, the sustained membrane current of CG cells is outwardly rectifying and loss of DPP6 reduces the size of this current (P = 0.008). The inset of Fig. 2C expands the membrane potential axis around the resting potential to better show that this change in the sustained current voltage relationship underlies the observed changes in membrane zero current potential (E_Rest_) and resting input resistance. A DPP6 dependent current was constructed by taking the difference between the average membrane current with and without DPP6 RNAi treatment (Fig. 2D). This analysis shows that the general shape of the I-V curve for the DPP6 dependent current is also outwardly rectifying, with a reversal near E_K_. Representative voltage clamp recordings from wild type control and mDPP6 RNAi treated CG cells show that the membrane conductance being regulated by DPP6 is rapidly outwardly rectifying, similar to the previously described I_K(SO)_ of CG cells (Fig. 2E) [8], [12].

### Pharmacology of DPP6 Dependent Current Matches TASK-3

**Figure 3:**
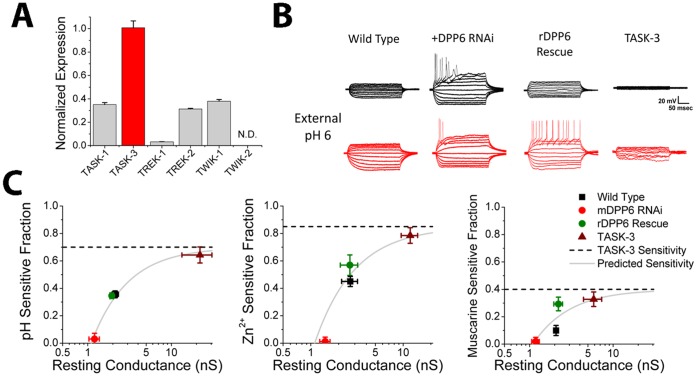
DPP6 regulates a channel with properties similar to TASK-3. **A)** SYBR-Green qRT-PCR on cDNA made from mRNA isolated from our CG cell cultures. Following primer optimization, expression levels were determined by the C_t_0.1 values (n = 6 for each). Five different K_2P_ channels could be detected with TASK-3 abundance about equal to the other four combined. TWIK-2 could not be detected (N.D.). (data plotted ± SEM). **B)** Sensitivity of membrane conductance to treatment with pH 6.0 external solution. Current clamp recordings from cells before and after application of pH 6.0 external solution show changes in the voltage responses to a series of 10 pA current steps. For these studies we only characterized changes in input conductance because pH 6.0 partially blocks voltage gated channels Na^+^ channels, making an analysis of effects of low pH on firing threshold complex and essentially uninformative for our purposes [73]. Control cells show much larger voltage responses following low pH application indicating that a significant fraction of the resting membrane conductance is pH sensitive. Treatment with mDPP6 RNAi decreases resting membrane conductance to levels similar to control CG cells treated with low pH while also eliminating sensitivity to low pH. Co-expression of rDPP6 restores sensitivity to low pH. TASK-3 driven by a CMV promoter was expressed in CG cells using a lentiviral vector to determine the sensitivity of TASK-3 to low pH in this expression system. As expected expression of high levels of TASK-3 dramatically increases membrane resting conductance, much of which is sensitive to block by external pH 6.0. Scale bar: 20 mV, 50 ms. **C)** Summary results testing sensitivity of the resting conductance to external pH 6.0 (WT (n = 11), mDPP6 RNAi (n = 5), rDPP6 Rescue (n = 8), TASK-3 (n = 6)), 500 µM external Zn^2+^ (WT (n = 6), mDPP6 RNAi (n = 5), rDPP6 Rescue (n = 6), TASK-3 (n = 6)) and suppression by 10 µM muscarine (WT (n = 8), mDPP6 RNAi (n = 5), rDPP6 Rescue (n = 4), TASK-3 (n = 6)). Results show that treatment with mDPP6 RNAi eliminates sensitivity to these blockers which is rescued by co-expression of rDPP6. Over expression of TASK-3 produced the highest sensitivity. Data points were well fit (solid line) assuming a single TASK-3 sensitivity (dashed line) for conductance levels above the 1.1 nS of average mDPP6 RNAi insensitive resting conductance in these experiments. (data plotted ± SEM).

In CG cells, previous studies have shown that K_2P_ channels play an important role in regulating resting membrane potential and input resistance and likely underlie I_K(SO)_ [9], [10], [12], [17]. To determine if a channel in this gene family is responsible for forming the DPP6 dependent current, qRT-PCR was performed on our CG cell cultures to measure the relative expression levels of different K_2P_ channels (Fig. 3A). qRT-PCR identifies TASK-3 as the most highly expressed K_2P_ subunit found in these neurons (P<0.0001). Since TASK-3 forms a rapidly outwardly rectifying potassium conductance, we hypothesized that TASK-3 was a likely candidate underlying the DPP6 dependent current [9], [10], and therefore tested if the conductance being regulated by DPP6 has pharmacology similar to that of TASK-3 channels. TASK channels are named for their sensitivity to block by external acidification, and we can pharmacologically distinguish between the related TASK-3 and TASK-1 proteins because only TASK-3 is highly sensitive to block by external Zn^2+^ [16], [17], [19], [35]. Fig. 3B shows representative voltage response of CG cells to a series of 10 pA current injections given before and after application of external pH 6.0 to examine the potential role of TASK channels in establishing the CG cells resting membrane conductance properties. In control cells, as expected for a significant TASK conductance, application of external pH 6.0 results in a large increase in amplitude of the elicited voltage deflections due to the block of a portion of the resting membrane conductance. However, in mDPP6 RNAi treated CG cells there is almost no effect of external pH 6.0 application. The loss of an acid sensitive conductance can be completely reversed by co-expression of RNAi resistant rDPP6, demonstrating that this effect is specifically due to the loss of DPP6. To directly compare the sensitivity of the DPP6 regulated conductance to TASK-3, we over expressed TASK-3 in CG cells and measured its pharmacological sensitivity. As expected, TASK-3 expression produces a dramatic increase in resting conductance that is sensitive to block by external pH 6.0.

Fig. 3C summarizes our results for block of the CG cell resting membrane conductance by external pH 6.0, 500 µM external Zn^2+^, and 10 µM muscarine following infection with various lentiviral constructs. For wild type CG cell, a significant fraction of the resting membrane conductance is sensitive to low pH, Zn^2+^ and Muscarine suggesting that TASK channels, and TASK-3 in particular, contribute significantly to the resting membrane properties of these cells. Following treatment with mDPP6 RNAi, the sensitivity of the resting membrane conductance to pH 6.0, external Zn^2+^, and muscarine is almost completely eliminated (Not significantly different from 0(P>0.5)), suggesting that the TASK component of the resting membrane conductance has been lost. The effects of mDPP6 RNAi and can be rescued back to wild type levels by co-expression of RNAi insensitive rDPP6 (compared to mDPP6 RNAi for each condition, P<0.0001). To determine the expected sensitivity of TASK-3 channels in our CG cells to these treatments, we over expressed TASK-3 using a lentiviral vector. As expected the pH, Zn^2+^ and muscarine sensitive components of the resting membrane conductance greatly increase as the levels of TASK-3 conductance increases. We used a simple model to fit these data where the resting membrane conductance of CG cells is assumed to be composed of two populations of channels, a DPP6 dependent TASK-3 channel and a DPP6 insensitive non-TASK K_2P_ channel. Using this model with two free parameters, the amplitude of non-TASK K_2P_ conductance (x-intercept), and the sensitivity of TASK-3 to the treatment (y-axis asymptote, dashed lines) we could explain the observed results (solid lines) (muscarine: r^2^ = 0.75; pH 6.0: r^2^ = 0.98, Zn^2+^: r^2^ = 0.87). Although DPP6 regulation of TASK-3 is sufficient to explain our results, we cannot rule out the importance of TASK-1/TASK-3 heteromultimers or the co-regulation of other K_2P_ channels that might require TASK-3 for functional expression or the potential regulation by DPP6 of other K_2P_ channels not expressed at high levels in CG cells.

### DPP6 Regulates TASK-3 in Heterologous Cells

**Figure 4:**
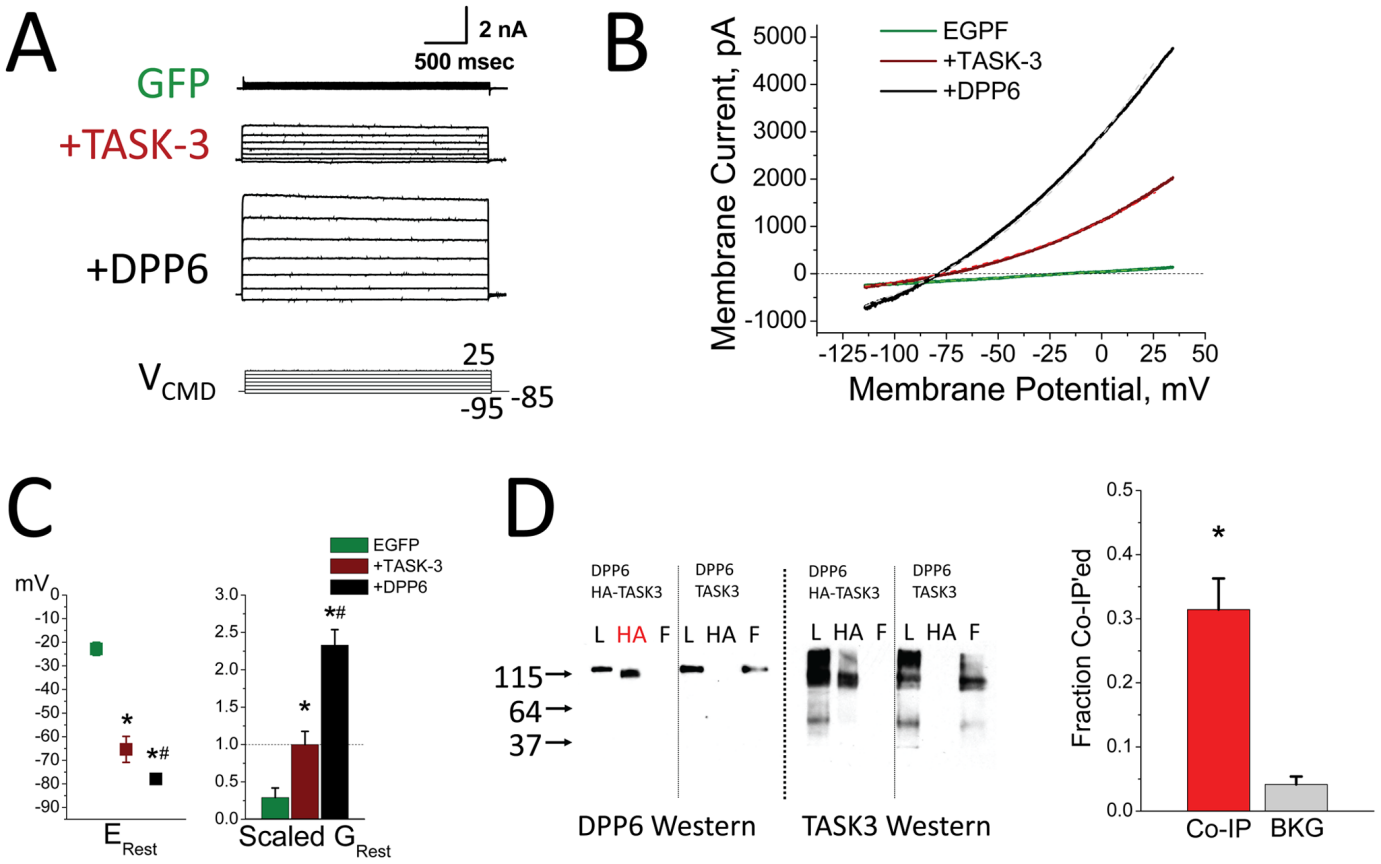
Regulation of TASK-3 by DPP6 in a Heterologous Expression System. CHO-K1 cells were transfected with GFP alone (GFP (n = 14)), or the addition of TASK-3 (+TASK-3 (n = 16)) or the further addition of DPP6 with TASK-3 (+DPP6 (n = 23)). A) Representative current traces for step depolarizations. Only small currents are observed in CHO cells that are not transfected with channel subunits. Following expression of TASK-3 there is a large outwardly rectifying current expressed that increases further with co-expression of DPP6. Scale bar: 2000 pA, 500 ms. B) Representative current-voltage plots for voltage ramps showing the outward rectifying potassium current produced by TASK-3 is greatly increased by addition of DPP6. Dashed lines are from a NEURON model incorporating a non-selective cationic Leak Channel (E_L_ = −16 mV) and a potassium selective TASK-3 Channel (E_K_ = −91.5 mV). Maximum conductance parameters used to generate these curves (EGFP: G_L_ = 2.81 nS, G_TASK-3_ = 0 nS; **+**TASK-3: G_L_ = 1.75 nS, G_TASK-3_ = 320 nS; **+**DPP6: G_L_ = 4.25 nS, G_TASK-3_ = 840 nS). C) Analysis of average changes in resting potential and resting membrane conductance. Membrane property changes produced by TASK-3 (*compared to GFP: E_Rest_(P<0.0001); G_Rest_(P<0.0036)) are further increased by co-expression of DPP6 (# compared to +TASK-3: E_Rest_ (P<0.023); G_Rest_ (P<0.0001)). (data plotted ± SEM). D) Co-immunoprecipitation was performed to test for direct interactions between DPP6 and TASK-3 followed by Western blots for either DPP6 or TASK-3. Expression conditions indicated at the top of each section. L-Load, HA-precipitated with anti-HA antibody, F-flow through. Red HA indicates a lane where the non-HA tagged protein has been co-immunoprecipitated with the HA-tagged protein. Summary data from 6 co-immunoprecipitation experiments shows that a co-precipitation protocol pulls down about 31.4±4.8% (P = 0.0013) of the protein that could be directly precipitated compared to a background signal of 4.0±1.3% when neither proteins is HA tagged (BKG).

To determine if DPP6 can specifically regulate TASK-3, we performed heterologous co-expression studies in CHO-K1 cells. CHO-K1 cells have a low resting membrane conductance and a depolarized resting membrane potential (Fig. 4A–C). Expression of TASK-3 produces a rapid outwardly rectifying potassium current (Fig. 4A, B), that hyperpolarizes the resting membrane potential of CHO-K1 cells and increases the resting membrane conductance (Fig. 4C). Co-expression of DPP6 with TASK-3 produces a dramatic increase in the magnitude of the rapid outwardly rectifying potassium current formed by TASK-3 alone (Fig. 4A, B). The addition of DPP6 further hyperpolarizes the resting membrane potential and leads to an additional 2.3 fold increase in the resting membrane conductance (Fig. 4C). To test for co-assembly of DPP6 and TASK-3 into a protein complex, co-immunoprecipitation experiments were performed. When DPP6 and TASK-3 are co-expressed with one protein epitope tagged with HA, they co-precipitate with about a third of the total protein co-precipitating on average, compared to a 4% background signal when neither protein is HA tagged (Fig. 4D). We conclude therefore that DPP6 can bind to a protein complex containing TASK-3 to regulate the level of functional current.

### Computational Modeling Shows DPP6 Co-Regulation of I_SA_ and I_K(SO)_ Tunes Neuronal Subthreshold Information Processing

**Figure 5:**
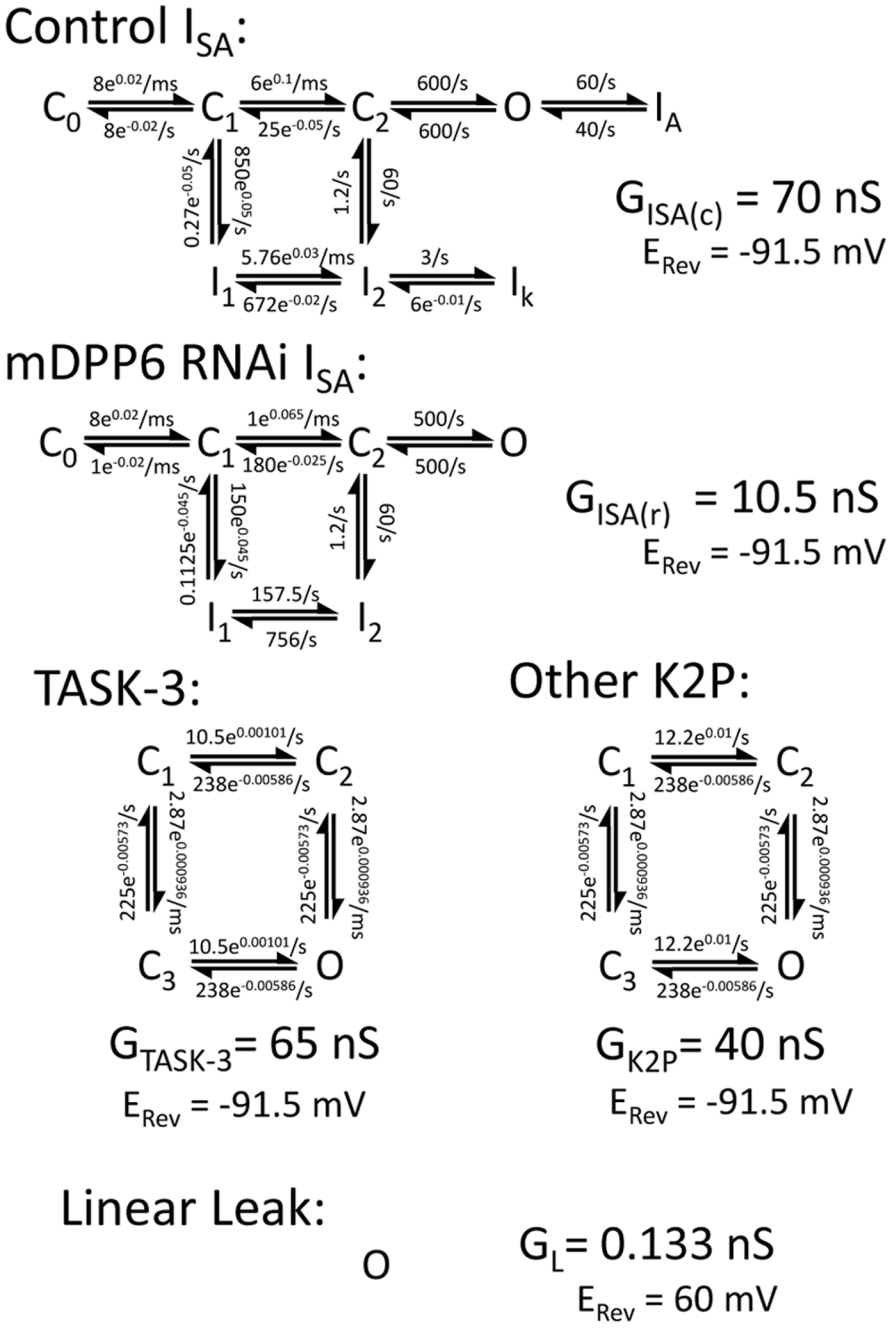
Conductance gating models for the different current components used in our CG cell NEURON model. Rate constants are k(0 mV) rate times the voltage dependence (e^a^) which is raised to the E_M_ power (mV) to obtain the final rate. G_max_ and 0 current potentials are given for each conductance. For I_SA_ models, G_ISA(c)_ and G_ISA(r)_, the I_1_−I_2_ transition between closed inactivated states is generally not rate limiting, and thus effectively invisible, but was adjusted to preserve detailed balances. More complex models have been proposed for I_SA_, but these models capture the basic gating properties of the I_SA_ channel over a wide range of voltages. For the TASK-3 channel, and the residual K_2P_ current in CG cells remaining after mDPP6 RNAi treatment, G_K2P_, the model has 2 independent gates which must both be open to conduct. The linear leak component is always open and modeled to be Na^+^ selective to match the relatively high input resistance of CG cells while still producing the large change in E_rest_ between Control and mDPP6 RNAi CG cells.

To examine the importance of co-regulation of both TASK-3 and I_SA_ channels by DPP6 in shaping CG cell firing and information processing, we constructed channel gating models using QuB for TASK-3(G_TASK-3_), mDPP6 RNAi insensitive K_2P_ (G_K2P_), control I_SA_ (G_ISA(c)_) and mDPP6 RNAi residual I_SA_ (G_ISA(r)_) and incorporated these channels as point processes into a single compartment (12.5 µm diameter) NEURON [22] CG cell model [6] (Fig. 5). Due to the electrotonically compact somatodendritic compartment in CG cells a multicompartment dendritic model is not necessary; however, accurately capturing some aspects of CG cell firing may require separate axonal and initial segment compartments that we did not try to model in this study [6], [36]. As described in the Methods, the G_ISA(c)_ and G_ISA(r)_ models were optimized to fit our recorded CG cell currents (Fig. S1). For G_TASK-3_ we used a model based on detailed single channel recordings of TASK-3 channels [27], and slightly modified it to match the conductance properties of the resting K_2P_ conductance, G_K2P_, found in mDPP6 RNAi treated CG cells (Fig. 5). The G_TASK-3_ and G_K2P_ models produce a good fit to our data from CG cells (Fig. 2C,D) and to TASK-3 currents expressed in CHO cells with or without DPP6 (Fig. 4B). By switching channel types present under different conditions (Control: G_TASK-3_, G_K2P_, G_ISA(c)_; mDPP6 RNAi: G_K2P_, G_ISA(r)_) the CG cell model accurately reproduces the resting membrane potential (Control E_Rest_ = −83.5 mV, mDPP6 RNAi E_Rest_ = −69.4 mV) and input resistance (Control R_IN_ = 0.49 GΩ, mDPP6 RNAi R_IN_ = 1.06 GΩ) for Control and mDPP6 RNAi treated CG cells. By doubling resting membrane conductance, DPP6 cuts the membrane time constant in half, increasing the frequency response properties of the CG cell membrane particularly in the gamma frequency range (f_c_: Control = 75 Hz, mDPP6 RNAi = 32 Hz).

**Figure 6:**
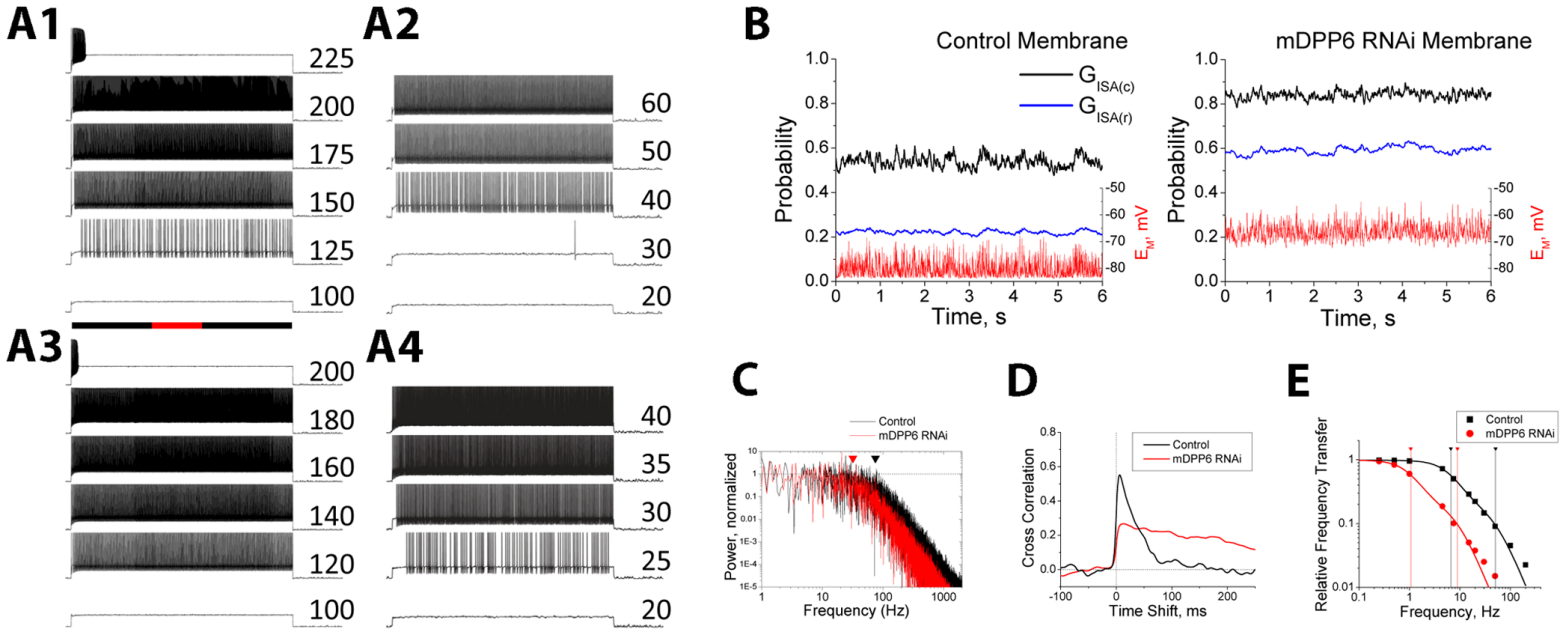
Co-regulation of Kv4 and TASK-3 channels by DPP6 optimizes the sensitivity of I_SA_ to membrane potential fluctuations. A) Firing properties of NEURON CG cell models. A1- Control, A2- CG(ΔTASK-3), A3- CG(I_SA(R)_), a4- mDPP6 RNAi. Current injection magnitude indicated to the right of the trace (pA). Black bar- injection duration, red inset- 1 s. A low level of random noise was added to these simulations (G_e_ = G_i_ = 10 pS; SD_e_ = SD_i_ = 50 pS). B) Effect of stochastic synaptic conductance fluctuations on I_SA_ inactivation (G_e_ = G_i_ = 25 pS; SD_e_ = SD_i_ = 200 pS). In Control CG model, membrane potential fluctuates around −81 mV. Control I_SA_ (G_ISA(c)_) responds with fluctuations in inactivation near the 0.5 level. For mDPP6 RNAi I_SA_ (G_ISA(r)_) inactivation would be much less (∼0.2). In the mDPP6 RNAi CG cell, the mDPP6 RNAi I_SA_ channel inactivation (G_ISA(r)_) is near the 0.5 level, whereas a control I_SA_ channel (G_ISA(c)_) would be around (0.8). G_ISA(c)_ inactivation show higher frequency components than G_ISA(r)_. C) Power spectra for membrane potential fluctuations in Control and mDPP6 RNAi membranes calculated by FFT using OriginPro. Calculated corner frequencies for Control and mDPP6 RNAi membranes are indicated by triangles. mDPP6 RNAi signals fall off before Control due to a difference in membrane time constant. D) Cross-correlation analysis for I_SA_ inactivation compared to membrane potential fluctuations. Control I_SA_ shows a peak correlation of around 0.55 at 6.4 ms delay to the Control membrane potential fluctuations. For mDPP6 RNAi I_SA_, the peak correlation is 0.27 at a 15 ms delay to the mDPP6 RNAi cell membrane potential fluctuations. E) Frequency response properties for I_SA_ channel inactivation. 5 mV sinusoidal waves from 0.25–200 Hz centered at −80 mV were fed into the G_ISA(c)_ and G_ISA(r)_ QuB channel models and the inactivation signal was measured and fit with a sine wave. The RMS amplitude of the fitted sine wave was compared to the inactivation produced by the RMS equivalent DC potential change of ±3.54 mV. The signal fall off at higher frequencies was fit with a double Lorentzian (corner frequencies indicated by drop lines). There is about a 7× shift to higher frequency responses when DPP6 is present in the I_SA_ channel.

We next examined the relative importance of I_SA_ and TASK-3 regulation for the changes in excitability produced by mDPP6 RNAi. When the firing of the Control CG cell model in response to current steps of varying amplitude (Fig. 6A1) is compared to the firing of the mDPP6 RNAi CG cell model (Fig. 6A4) the shift in threshold current is very similar to what we see in our recordings (Model: 93 pA; Recordings: 96±13.6 pA). To determine the relative importance of I_SA_ and TASK-3 modulation for producing these changes in firing, we selectively reproduced only the change in I_SA_ produced by mDPP6 RNAi (CG(I_SA(R)_): G_TASK-3_, G_K2P_, G_ISA(r)_) (Fig. 6A2) or only the loss of TASK-3 produced by mDPP6 RNAi (CG(ΔTASK-3): G_K2P_, G_ISA(c)_) (Fig. 6A4). Fig. 6A2 shows that in the CG(ΔTASK-3) model, the selective loss of TASK-3 reduces threshold current by 80 pA, suggesting that most of the increase in excitability produced by mDPP6 RNAi is due to the loss of this channel. The larger amplitude and more rapid kinetics of the Control I_SA_ is evident by the strong suppression of firing early in the depolarization. In contrast, the CG(I_SA(R)_) model (Fig. 6A3) shows only a 12 pA decrease in threshold current, but much less suppression of firing early in the depolarization compared to Control.

In addition to direct effects on excitability, co-regulation of Kv4 and TASK-3 channels by DPP6 has important implications for how CG cells processes subthreshold membrane potential fluctuations. I_SA_ channel inactivation changes dramatically in the subthreshold range of membrane potentials, and is thought to be an important short term associative learning signal [28], [37], [38], [39], [40]. DPP6 greatly accelerates I_SA_ activation and inactivation kinetics [6], complementing its effects on membrane frequency response. We therefore sought to use our models to examine how mDPP6 RNAi treatment might affect the translation of subthreshold membrane potential fluctuations into different levels of I_SA_ channel inactivation. For these studies we used a stochastic signal generator to introduce excitatory and inhibitory conductance fluctuations to alter membrane potential around rest [23] (Figure 6B). The amplitudes of current fluctuations were adjusted to produce an average RMS fluctuation of around 2.8 mV in the resting membrane potential for Control (G_e_ = G_i_ = 25 pS; SD_e_ = SD_i_ = 200 pS) and mDPP6 RNAi conditions (G_e_ = G_i_ = 25 pS; SD_e_ = SD_i_ = 110 pS). Due to the lower membrane corner frequency (f_c_) in mDPP6 RNAi CG cells, there is less power in these fluctuations at higher frequencies compared to Control (Fig. 6C). For Control and mDPP6 RNAi CG cell models, recorded membrane potential fluctuations were used as input to I_SA_ channel models in QuB to determine the impact on inactivation (Figure 6B). On average, due to coordinate shifts in CG cell resting membrane potential and I_SA_ gating produced by DPP6, I_SA_ steady state inactivation is positioned near the midpoint at rest under both conditions, at the steepest point of the inactivation curve. In contrast, for G_ISA(c)_ responses to the mDPP6 RNAi membrane, or G_ISA(r)_ responses to the Control membrane, the resting inactivation level of the channel are at one extreme end or the other of the inactivation curve. Although the average values for inactivation are similar for I_SA_ in Control or mDPP6 RNAi treated CG cells, the sensitivity of inactivation to the E_M_ fluctuations is much greater under Control conditions as can be seen by comparing the standard deviation of the resultant inactivation fluctuations to the standard deviation of the input E_M_ fluctuations (SD ratios: Control: 0.9%/mV; mDPP6 RNAi: 0.57%/mV).

To quantify how reliably I_SA_ channels convert membrane potential fluctuations into changes in inactivation, we measured the cross-correlation between inactivation and membrane potential (Fig. 6D). For Control conditions, the peak correlation of I_SA_ channel inactivation with membrane potential is 0.55 at a delay of 6.3 ms. This correlation is not seen in the forward direction and falls off rapidly with a time constant of 35 ms. In contrast, the peak correlation of mDPP6 RNAi I_SA_ channel inactivation with membrane potential is much lower (0.27), peaks later (15 ms), and falls off more slowly with a time constant of 300 ms. We also examined a range of stochastic input parameters (G_e_,G_i_: 0–50 pS; SD_e_,SD_i_: 10–500 pS) and membrane potential offsets, and find similar results. The shape of the cross-correlation plot for the Control condition is strikingly similar to the time course for spike timing dependent plasticity, suggesting that DPP6 expression is likely important in regulating this plasticity signal [41]. To directly compare the frequency responses of the Control and mDPP6 RNAi I_SA_ channels we introduced 5 mV sinusoidal membrane potential fluctuations from 0.25–200 Hz around −80 mV into our QuB I_SA_ models and measured how efficiently these fluctuations were converted into changes in inactivation (Fig. 6E). Models incorporating control I_SA_ channels respond to much higher frequency components, suggesting that the higher frequencies membrane potential fluctuations allowed by DPP6 regulation of TASK-3 channels can only be effectively utilized by I_SA_ channels incorporating DPP6. Taking all these effects together suggests that DPP6 co-regulation serves to facilitate the efficient translation of dynamic fluctuations in membrane potential into significant changes in the level of available I_SA_.

## Discussion

The idea of ion channel proteins as isolated functional entities has long since been overturned. It is now clear that most channel proteins are complex multipeptide machines that interact with a variety of other cellular components to properly localize, functionally shape, and regulate the ion currents they pass. Recently some integral proteins of ion channel complexes have been shown to have multiple distinct functional roles in a cell. For example, calmodulin plays an integral role in many channels and enzymes, Na^+^ channel auxiliary subunits have also been identified as important components of voltage-gated potassium channels, KChIPs have been shown to regulate the function of both voltage-gated calcium channels and potassium channels as well as regulate gene transcription, and KCNE1-5 can regulate both KCNQ channels as well as HERG channels and likely other potassium channels [42], [43], [44], [45], [46], [47]. While it is easy to envision why the efficient Ca^2+^ sensing of calmodulin has been co-opted to regulate a number of different protein complexes, including ion channels, it is less clear why so many ion channel auxiliary subunit protein appear to have multiple functional roles.

Prior to these studies the only known role for DPP6 was to regulate Kv4 channel expression and functional properties, and we hypothesized that this regulation was critical to form the native somatodendritic A-type current (I_SA_) in neurons [3], [5], [7], [34], [48], [49], [50], [51]. In this study, we add DPP6 to this growing list of multifunctional channel auxiliary subunit proteins by showing it can also co-regulate a resting conductance with functional properties that are similar to TASK-3 channels. Our computational modeling studies suggest a potential new functional role for DPP6 by considering all the physiological changes regulated by DPP6 as part of a whole. In this view, it is clear that DPP6 can be seen to play an important role in speeding up CG cell electrophysiological properties by accelerating channel gating kinetics and increasing the cellular membrane potential frequency response. This suggests an innovative hypothesis, namely that DPP6 has evolved to produce a coordinate regulation of multiple ion channels to produce a dramatic overall change in neuronal functional phenotype. Interestingly, KChIP regulation of voltage-gated Ca^2+^ channels and Kv4 channels has a related integrative functional role in shaping the excitability of stellate cells [52], [53]. It therefore seems likely that many auxiliary subunits may be playing similar integrative functional roles by interacting with multiple channel types to shape the excitability properties of neurons and other excitable cells.

While computational modeling clearly suggests an integrative function for DPP6 in shaping CG cell processing of synaptic inputs and firing properties, more work needs to be done to fully establish such a role *in vivo*. In addition to potential differences in gene expression and protein targeting *in vivo*, other regulatory factors such as GABA inhibition have not yet been incorporated into our model [54]. Indeed, precise *in vivo* patterns of mossy fiber release onto CG cells have not been modeled in our studies, and therefore we can only generally describe how DPP6 shapes the frequency response properties of CG cells without being able to better describe when such frequencies are important for cerebellar function. In addition, there are aspects of compartmentalized neuronal function that we have not attempted to capture in our CG cell model. CG cells do not have the large complex dendritic trees of CA1 and cortical pyramidal neurons, although DPP6 does regulate potassium channels in such dendrites and therefore DPP6 co-regulation may play an important role in shaping dendritic tree function in other neurons [51]. Finally, there are important inter-compartment interactions in CG cells between the somatodendritic and axon and axon hillock regions that we have not modeled and may play an important role in regulating CG cell firing *in vivo* [36]. For the most part, however, incorporating such additional factors and caveats will likely only further refine the overall model that we have outlined here.

DPP6 is a large multi-domain protein so it is perhaps not surprising that it has other functional roles in the nervous system beyond regulation of I_SA_. DPP6 is closely related to another inactive protease DPP10, and together they make up the DPL (dipeptidyl peptidase-like) gene family. Because DPP10 is not expressed in CG cells, our studies have focused on DPP6; however, it is now clear that we need to rethink this gene family in light of our new observations here. While we have identified many ways *in vitro* in which DPP6 can alter Kv4 channel expression and function, the exact importance of any given mechanism *in vivo* is less clear [3], [4], [26], [31], [50], [55], [56], [57]. With regards to K_2P_ channels, additional experiments are in order. Although knockdown of DPP6 dramatically lowers Kv4 and K_2P_ currents, it is possible that in CG cells DPP6 expression level is not normally a type of “master rheostat” regulating the level of potassium channel functional expression. For one thing, rescue of mDPP6 RNAi effects by DPP6 over expression returns I_SA_ and I_K(SO)_ back to their normal level, suggesting some other factors normally control the levels of these currents [6]. Indeed, we find that over expression of TASK-3 greatly increases the resting membrane conductance suggesting the resting level of I_K(SO)_ is limited by K_2P_ channel expression and not the level of DPP6. Thus, it seems likely that in CG cell DPP6 expression is not normally limiting, although it is possible that over expressed TASK-3 escapes normal controls on expression level.

Multiple distinct N-terminal variants of DPP6 are produced in CG cells through the use of alternative promoters in the DPP6 gene [5]. Heterologous expression studies show that these DPP6 N-terminal variants differentially regulate Kv4 channel function, providing an alternative mechanism for DPP6 to differentially regulate neuronal function [5], [26], [33], [34], [58]. In this study we have found differential effects of DPP6a and DPP6K on the resting membrane potential and input resistance of CG cells (see Fig. 2A,B). Future studies will need to determine the importance of differential DPP6 variant expression in shaping neuronal excitability properties of CG cells and other neurons.

Several recent studies have emphasized the potential links between DPL genes and human disease processes. Human genetic linkage studies have linked DPL genetic variations to asthma, amyotrophic lateral sclerosis, ventricular fibrillations, cancer, craniofacial disorders, and autism spectrum disorders [59], [60], [61], [62], [63], [64], [65], [66], [67], [68]. Interestingly, some of these disorders, including cancer, facial dysmorphisms, and mental retardation have been linked to K_2P_ channels but not Kv4 channels [69], [70], [71]. Whether these diseases are caused by DPL dysregulation of K_2P_ or Kv4 channels or some other as yet unidentified function of DPL proteins is not known. However, it is now clear that a better understanding of the roles of DPL proteins in health and disease will require a more complete understanding of how DPL proteins function beyond just the regulation of Kv4 channels.

Finally, as part of a functional network, neurons must strike a balance between relaying and retaining incoming information. Our studies suggest that in CG cells DPP6 expression pushes this balance towards more retaining and internal processing of incoming information. In the absence of DPP6, CG cells are much more likely to relay information since the neuron reaches threshold more easily in response to excitatory inputs and thus most input spikes will generate output spikes with a minimal change in the information content. In the presence of DPP6, the same inputs are more likely to be retained and encoded as dynamic changes in the inactivation state of I_SA_ channels with only the strongest inputs producing action potential firing. Thus the incoming information is transformed into patterns of I_SA_ channel inactivation in a way that is tuned by DPP6 effects on I_SA_ channel steady state and kinetic gating properties as well as effects on resting membrane properties. Ultimately these changes in I_SA_ channel gating will dynamically affect the function of the entire network by altering neuronal excitability, and synaptic strengths by regulating action potential backpropagation [51], [72]. It will be interesting in future studies *in vivo* to determine how changes in the expression patterns for DPP6, and DPP6 variants, affect how neurons process information, and whether these effects are modulated by the differential expression of other channel components.

## Supporting Information

Figure S1 **Fits of I_SA_ gating models to the Data.** Currents for I_SA_ models under Control (G_ISA(c)_) and mDPP6 RNAi (G_ISA(r)_) treatment conditions are compared to our published properties for CG cell I_SA_ recorded under the same conditions [6]. Symbols are data taken from our published CG cell recordings. Solid curves (B, C, D) were generated by our G_ISA(c)_ and G_ISA(r)_ models. A) Representative currents in response to a step to 6 mV. Symbols- average peak current recorded from CG cells under these conditions. Model currents (black) are compared to recorded currents (red) (recorded currents scaled to facilitate comparison of inactivation kinetics). B) Fits to I_SA_ steady state inactivation and peak activation data. C) Recovery data for CG cell I_SA_. D) Time to peak data for CG cell I_SA_. E) Histogram showing distribution of fast inactivation taus recorded with strong depolarizations >40 mV. Vertical lines show the measured fast inactivation taus for our G_ISA(c)_ and G_ISA(r)_ models at 50 mV.
(TIF)
