# Supplementary figures and images for: A New TASK for Dipeptidyl Peptidase-like Protein 6
Source: PLoS One. 2013 Apr 9;8(4):e60831. doi: 10.1371/journal.pone.0060831 (PMC3621905; doi:10.1371/journal.pone.0060831)

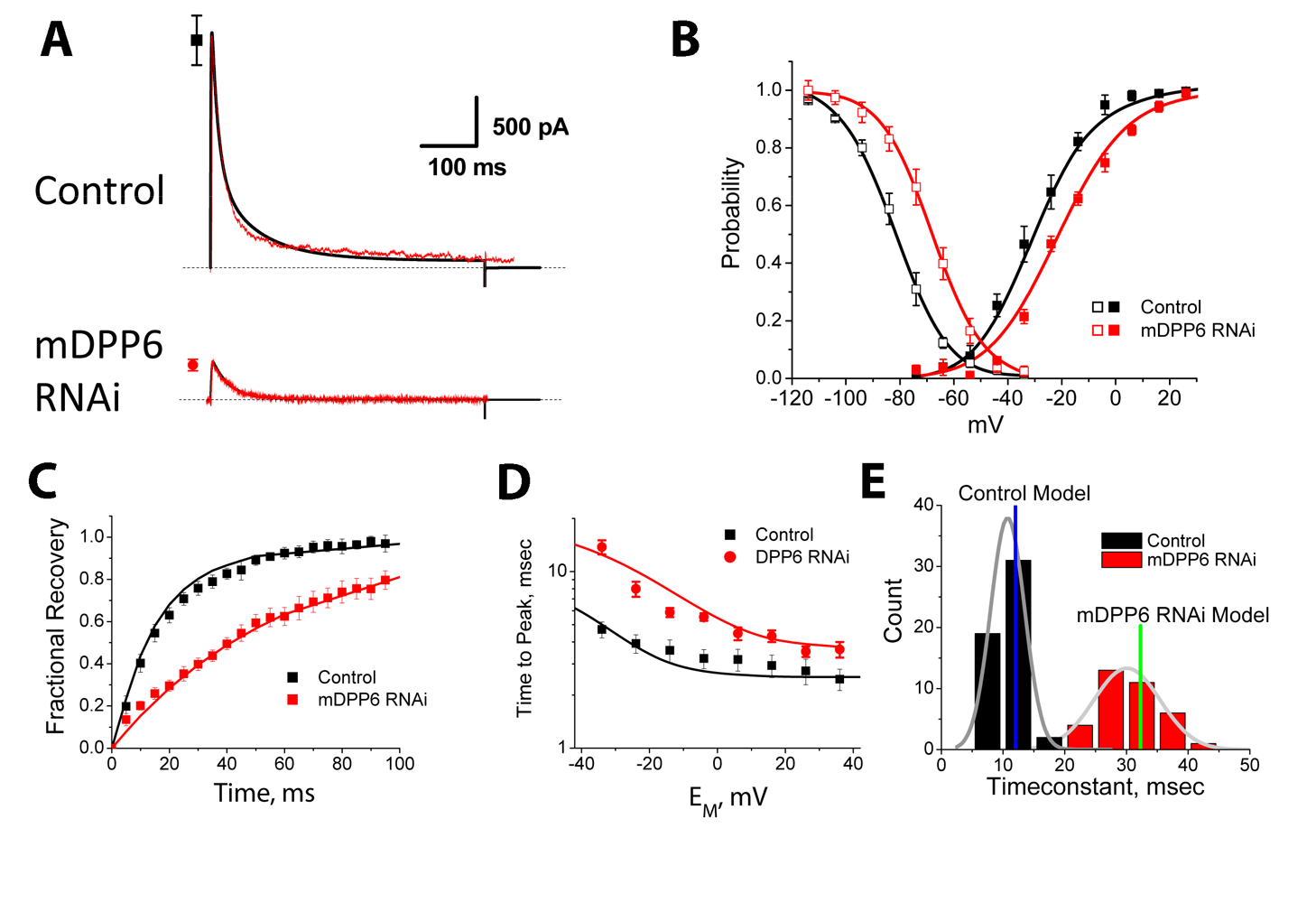

Supplement: Figure S1 — Fits of ISA gating models to the Data. Currents for ISA models under Control (GISA(c)) and mDPP6 RNAi (GISA(r)) treatment conditions are compared to our published properties for CG cell ISA recorded under the same conditions [6]. Symbols are data taken from our published CG cell recordings. Solid curves (B, C, D) were generated by our GISA(c) and GISA(r) models. A) Representative currents in response to a step to 6 mV. Symbols- average peak current recorded from CG cells under these conditions. Model currents (black) are compared to recorded currents (red) (recorded currents scaled to facilitate comparison of inactivation kinetics). B) Fits to ISA steady state inactivation and peak activation data. C) Recovery data for CG cell ISA. D) Time to peak data for CG cell ISA. E) Histogram showing distribution of fast inactivation taus recorded with strong depolarizations >40 mV. Vertical lines show the measured fast inactivation taus for our GISA(c) and GISA(r) models at 50 mV. (TIF) [file pone.0060831.s001.tif]
